# *Staphylococcus aureus* bloodstream infections: diverging trends of meticillin-resistant and meticillin-susceptible isolates, EU/EEA, 2005 to 2018

**DOI:** 10.2807/1560-7917.ES.2021.26.46.2002094

**Published:** 2021-11-18

**Authors:** Carlo Gagliotti, Liselotte Diaz Högberg, Hanna Billström, Tim Eckmanns, Christian G Giske, Ole E Heuer, Vincent Jarlier, Gunnar Kahlmeter, Danilo Lo Fo Wong, Jos Monen, Stephen Murchan, Gunnar Skov Simonsen, Maja Šubelj, Arjana Tambić Andrašević, Dorota Żabicka, Helena Žemličková, Dominique L Monnet, EARS-Net study group participants, EARS-Net study group participants, Reinhild Strauss, Lucy Catteau, Yuliya Stoyanova Marteva-Proevska, Silvija Soprek, Panagiota Maikanti-Charalampous, Vladislav Jakubů, Andreas Petersen, Marina Ivanova, Laura Lindholm, Sylvie Maugat, Ines Noll, Michalis Polemis, Zsolt Végh, Karl Gústaf Kristinsson, Karen Burns, Monica Monaco, Ieva Rutkovska, Jolanta Miciulevicienė, Monique Perrin, Elizabeth A. Scicluna, Sjoukje H. S. Woudt, Frode Width Gran, Waleria Hryniewicz, Manuela Caniça, Gabriel Adrian Popescu, Milan Niks, Helena Ribič, Maria Belén Aracil García, Barbro Mäkitalo, Russell Hope

**Affiliations:** 1 Regional Agency for Health and Social Care of Emilia-Romagna, Bologna, Italy; 2 European Centre for Disease Prevention and Control, Solna, Sweden; 3 Public Health Agency of Sweden, Solna, Sweden; 4 Healthcare-associated infections, surveillance of antimicrobial resistance and consumption, Department for Infectious Disease Epidemiology, Robert Koch Institute, Berlin, Germany; 5 Division of Clinical Microbiology, Department of Laboratory medicine, Karolinska Institutet and Karolinska University Hospital, Stockholm, Sweden; 6 Sorbonne Universités (Paris 06) Inserm Centre d'Immunologie et des Maladies Infectieuses (CIMI), UMR 1135 & APHP, Pitié-Salpêtrière hospital, Laboratoire de Bactériologie-Hygiène, Paris, France; 7 Clinical microbiology, Växjö Central hospital, Växjö, Sweden; 8 World Health Organization, Regional Office for Europe, Copenhagen, Denmark; 9 National Institute for Public Health and the Environment, Bilthoven, The Netherlands; 10 Health Protection Surveillance Centre, Dublin, Ireland; 11 Department of Microbiology and Infection Control, University Hospital of North Norway, Tromsø, Norway; 12 Research Group for Host Microbe Interaction, Faculty of Health Sciences, UiT – The Arctic University of Norway, Tromsø, Norway; 13 Norwegian Institute of Public Health, Oslo, Norway; 14 National Institute of Public Health, University of Ljubljana, Slovenia; 15 Zagreb University Hospital for Infectious Diseases, Zagreb, Croatia; 16 Department of Epidemiology and Clinical Microbiology, National Medicines Institute, Warsaw, Poland; 17 National reference laboratory for antibiotics, National Institute of Public Health, Prague, Czech Republic; 18 Department of Microbiology, 3rd Faculty of Medicine Charles University, University hospital Kralovske Vinohrady, and National Institute of Public Health, Prague, Czech Republic; 19 EARS-Net study group participants are listed under Investigators

**Keywords:** *Staphylococcus aureus*, MRSA, Europe, antimicrobial resistance, bacterial infections, bloodstream infection

## Abstract

**Background:**

Invasive infections caused by *Staphylococcus aureus* have high clinical and epidemiological relevance. It is therefore important to monitor the *S. aureus* trends using suitable methods.

**Aim:**

The study aimed to describe the trends of bloodstream infections (BSI) caused by meticillin-resistant *S. aureus* (MRSA) and meticillin-susceptible *S. aureus* (MSSA) in the European Union (EU) and the European Economic Area (EEA).

**Methods:**

Annual data on *S. aureus* BSI from 2005 to 2018 were obtained from the European Antimicrobial Resistance Surveillance Network (EARS-Net). Trends of BSI were assessed at the EU/EEA level by adjusting for blood culture set rate (number of blood culture sets per 1,000 days of hospitalisation) and stratification by patient characteristics.

**Results:**

Considering a fixed cohort of laboratories consistently reporting data over the entire study period, MRSA percentages among *S. aureus* BSI decreased from 30.2% in 2005 to 16.3% in 2018. Concurrently, the total number of BSI caused by *S. aureus* increased by 57%, MSSA BSI increased by 84% and MRSA BSI decreased by 31%. All these trends were statistically significant (p < 0.001).

**Conclusions:**

The results indicate an increasing health burden of MSSA BSI in the EU/EEA despite a significant decrease in the MRSA percentage. These findings highlight the importance of monitoring antimicrobial resistance trends by assessing not only resistance percentages but also the incidence of infections. Further research is needed on the factors associated with the observed trends and on their attributable risk.

## Introduction

*Staphylococcus aureus* bloodstream infection (BSI) has high clinical relevance and is a major public health challenge [1]. Meticillin-resistant *S. aureus* (MRSA) complicates treatment of severe infections, causing increased morbidity, mortality and additional costs, and contributes to a large proportion of the antimicrobial resistance (AMR) burden in Europe [2,3].

During the past decade, the percentage of MRSA BSI among all *S. aureus* BSI reported to the European Antimicrobial Resistance Surveillance Network (EARS-Net) decreased in most European Union (EU) and European Economic Area (EEA) countries [4]. However, a recent European Centre for Disease Prevention and Control (ECDC) study on the health burden of AMR in Europe showed that the incidence of MRSA BSI had increased between 2007 to 2015, while confirming that the MRSA percentage had decreased significantly during the same period [2].

Previous studies have suggested that MRSA causes additional infections rather than replaces infections caused by meticillin-susceptible *S. aureus* (MSSA) [5-7], and that MRSA and MSSA trends can differ depending on the site of infection and population characteristics [8-11]. Nevertheless, these patterns are usually not routinely analysed by international surveillance systems, including EARS-Net, which generally focus on the percentage of *S. aureus* that are MRSA. It is, however, possible that changes in the incidence of MRSA and of MSSA infections exist and go unnoticed when only the MRSA percentage out of the total of *S. aureus* infections is assessed [6].

To better understand the relationship between MRSA BSI and MSSA BSI in the EU/EEA, we analysed the trends in the numbers and proportions of MRSA and MSSA BSI cases available from EARS-Net, by patient characteristics and in the context of potential changes in the frequency of using blood cultures for diagnostic purposes, between 2005 and 2018.

## Methods

### Data source and inclusion criteria

The EARS-Net is a surveillance network that collects routine clinical antimicrobial susceptibility testing (AST) results and some patient data from EU and EEA countries. The AST results are ascertained according to agreed protocols [12,13], and the general quality and comparability of the data are evaluated through an annual external quality assessment exercise offered to the participating laboratories [14]. The network has been coordinated by ECDC since 2010.

Data on *S. aureus* BSI reported to EARSS/EARS-Net for the period 2005 to 2018 were extracted from The European Surveillance System (TESSy) database at ECDC.

Resistance to meticillin in *S. aureus* isolates was determined by the reporting laboratories by phenotypic susceptibility tests, molecular methods or the latex agglutination test, as specified in the EARS-Net reporting protocol [12], and BSI episodes were classified as either MRSA BSI or MSSA BSI. Data were de-duplicated taking only the first *S. aureus* BSI per patient and year into account.

Data were stratified by patient age group (< 1 year, 1–24 years, 25–49 years, 50–64 years, 65–79 years and ≥ 80 years), sex (male or female) and hospital unit (intensive care unit (ICU) or other inpatient hospital unit). The stratum ICU included patients in adult ICU, paediatric ICU and neonatal ICU. A separate analysis was performed for outpatients, defined as patients not admitted to a hospital at the time of sampling of blood cultures.

We used two datasets for the analyses: dataset A included all BSI reported during the study period, while dataset B only included BSI from laboratories that reported data consistently for all years during the period 2005 to 2018. The purpose of dataset B was to have a fixed cohort of laboratories for studying the trends in the percentages of MRSA and the number of MRSA and MSSA BSI. Surveillance data before 2005 were excluded because of low numbers of laboratories and reported BSI.

For each country and year, a blood culture set rate, defined as the number of blood culture sets per 1,000 days of hospitalisation, was extracted from TESSy. We included these data in the analysis as blood culture sampling practices may have changed during the study period as a result of increased awareness of the health impact of sepsis [15]. The blood culture set rate was therefore considered as a potential confounder in the evaluation of the BSI trends.

### Analysis

Annual EU/EEA crude and population-weighted MRSA percentages were calculated for both dataset A and B in order to ascertain representativeness of dataset B. The EU/EEA population-weighted mean MRSA percentage was determined using as weights the population proportions of each country over the total population of the included countries. Annual population data were retrieved from the Eurostat online database [16].

For both datasets A and B, we calculated the ratio between the numbers of MSSA BSI and MRSA BSI in each year. Based on dataset B, we carried out further analyses, stratified by age category, sex and hospital unit and for outpatients, on the percentage of MRSA BSI out of reported *S. aureus* BSI, the number of MRSA and MSSA BSI and their annual variation using 2005 data as a baseline. For the stratified analyses, cases with missing information for the strata of interest were excluded.

Annual EU/EEA weighted means of blood culture set rates were calculated for dataset B. The country proportions of *S. aureus* BSI cases over the total cases of dataset B were used as weights. For dealing with missing data, country rates from the previous or following year were used, if available. When these were not available, the blood culture set rate was considered missing and the concerned countries were not considered in the calculation of the EU/EEA weighted mean. To check for the impact of these exclusions, we conducted a sensitivity analysis by selecting the countries for which the rates were estimated for all the years. The effect of the annual blood culture set rate as a potential confounder of the temporal trends of the number of *S. aureus*, MSSA and MRSA BSI was analysed.

The statistical significance of MRSA percentage trends over time was tested by a chi-squared test for a linear trend. A Poisson regression model was used for testing the statistical significance of trends of the numbers of MRSA and MSSA BSI. A significance level of 0.05 was considered in the study. Stata Statistical Software (StataCorp. 2015. Stata Statistical Software: Release 14. College Station, TX: StataCorp LP) was used for the analysis.

## Ethical statement

This study only included anonymised surveillance data; therefore, ethical approval was not required.

## Results

Between 2005 and 2018, a total of 573,951 *S. aureus* BSI cases (96,918 MRSA and 477,033 MSSA) were reported to EARS-Net from 1,295 laboratories in 30 countries (dataset A) (Supplementary Table S1). The number of BSI cases reported per year increased from 27,125 in 2005 to 72,085 in 2018 (Table). When restricted to only laboratories that continuously reported data during 2005 to 2018, 258,488 BSI cases (41,033 MRSA and 217,415 MSSA) were reported from 285 laboratories in 25 countries (dataset B, with data from all countries that reported data to EARS-Net except Croatia, Germany, Lithuania, Poland and Slovakia) (Supplementary Tables S1 and S2). The number of *S. aureus* BSI cases increased from 15,192 in 2005 to 23,879 in 2018 (Table).

**Table:** Bloodstream infections caused by meticillin-resistant (n = 96,918) and meticillin-susceptible *Staphylococcus aureus* (n = 477,033), number of reporting laboratories and countries, EU/EEA, 2005–2018

Year	Countries	Laboratories	*Staphylococcus aureus* BSI					
			Total	MRSA			MSSA	
	n	n	n	n	Crude %	Population-weighted %	n	
All laboratories (dataset A)								
2005	29	664	27,125	6,615	24.4	28.6	20,510	
2006	29	650	28,287	6,551	23.2	27.3	21,736	
2007	29	643	30,027	6,605	22.0	23.6	23,422	
2008	29	637	29,339	5,981	20.4	23.5	23,358	
2009	28	643	30,729	5,969	19.4	23.5	24,760	
2010	29	673	32,961	6,149	18.7	22.6	26,812	
2011	30	685	35,712	5,863	16.4	21.8	29,849	
2012	30	714	36,988	6,019	16.3	21.5	30,969	
2013	30	725	40,968	6,673	16.3	20.5	34,295	
2014	30	714	40,906	6,545	16.0	19.6	34,361	
2015	30	738	45,509	7,313	16.1	19.0	38,196	
2016	30	805	57,387	8,005	13.9	17.7	49,382	
2017	30	853	65,928	8,500	12.9	16.8	57,428	
2018	30	884	72,085	10,130	14.1	16.4	61,955	
Laboratories that consistently reported data (dataset B)								
2005	25	285	15,192	3,570	23.5	30.2		11,622
2006	25	285	15,954	3,617	22.7	28.8		12,337
2007	25	285	16,761	3,787	22.6	25.6		12,974
2008	25	285	16,307	3,253	19.9	24.1		13,054
2009	25	285	16,232	3,069	18.9	25.1		13,163
2010	25	285	15,987	2,868	17.9	23.0		13,119
2011	25	285	17,229	2,673	15.5	20.7		14,556
2012	25	285	17,622	2,740	15.5	22.8		14,882
2013	25	285	19,507	2,587	13.3	20.0		16,920
2014	25	285	18,833	2,573	13.7	20.3		16,260
2015	25	285	20,432	2,692	13.2	18.9		17,740
2016	25	285	21,711	2,547	11.7	17.6		19,164
2017	25	285	22,802	2,577	11.3	17.0		20,225
2018	25	285	23,879	2,480	10.4	16.3		21,399

BSI: bloodstream infections; EEA: European Economic Area; EU: European Union; MRSA: meticillin-resistant *Staphylococcus aureus*; MSSA: meticillin-susceptible *S. aureus*.

Results from all laboratories, 30 EU/EEA countries (dataset A) and from laboratories in 25 EU/EEA countries, which consistently reported data during the study period (dataset B).

The weighted average rate of blood culture sets for the countries included in dataset B increased notably from 35.8 per 1,000 hospital patient days in 2005 to 81.7 in 2018 (Supplementary Table S3). The sensitivity analysis conducted by selecting only the countries for which the blood culture set rates were estimated for all the years 2005 to 2018 (13/25 countries) showed a temporal trend of blood culture set rates from 33.1 per 1,000 hospital patient days in 2005 to 71.8 in 2018, comparable to that observed for the entire dataset B (Supplementary Table S3).

### Overall trends

Statistically significant decreasing trends in the MRSA percentage were observed for both the crude and the population-weighted EU/EEA means, and in both dataset A and B (Table). The crude MRSA percentage decreased significantly from 24.4% in 2005 to 14.1% in 2018 (p < 0.001) based on results of all laboratories (dataset A) and from 23.5% in 2005 to 10.4% in 2018 (p < 0.001) for consistently reporting laboratories (dataset B). The EU/EEA population-weighted MRSA percentage significantly decreased from 28.6% in 2005 to 16.4% in 2018 (p < 0.001) for all laboratories (dataset A) and from 30.2% in 2005 to 16.3% in 2018 (p < 0.001) based on consistently reporting laboratories (dataset B). The MSSA-to-MRSA ratio increased from 3.1 to 6.1 for dataset A and from 3.3 to 8.6 for dataset B.

**Figure 1:**
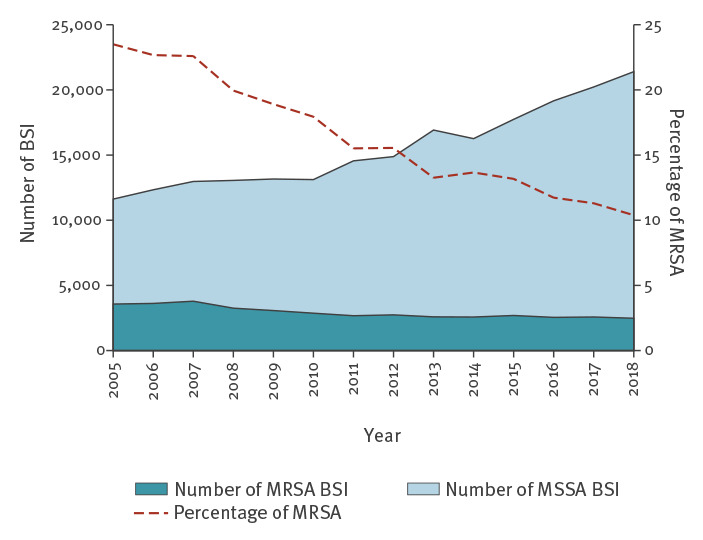
Bloodstream infections caused by meticillin-resistant and meticillin-susceptible *Staphylococcus aureus*, EU/EEA, 2005–2018 (n = 258,448)

BSI: bloodstream infections; EEA: European Economic Area; EU: European Union; MRSA: meticillin-resistant *Staphylococcus aureus*; MSSA: meticillin-susceptible *S. aureus*.

For dataset B, the total number of *S. aureus* BSI increased by 57% from 2005 to 2018, with a significant trend (p < 0.001). This general increase was the result of an 84% increase in the number of MSSA BSI cases and a 31% decrease in the number of MRSA BSI cases (Table; Figure 1). Based on the Poisson model, the mean annual crude changes in the number of cases were +3.5% for *S. aureus* BSI, +4.8% for MSSA BSI and −3.3% MRSA BSI. After adjusting for the blood culture set rate, these changes were +1.0%, +2.9% and −5.6%, respectively. All these trends were statistically significant (p < 0.001).

Results from 285 laboratories in 25 EU/EEA countries, which consistently reported data during 2005 to 2018 (dataset B).

### Trends by patient characteristics

Trend analyses by patient characteristics were restricted to dataset B in order to study a stable cohort of laboratories and a defined population under surveillance.

**Figure 2:**
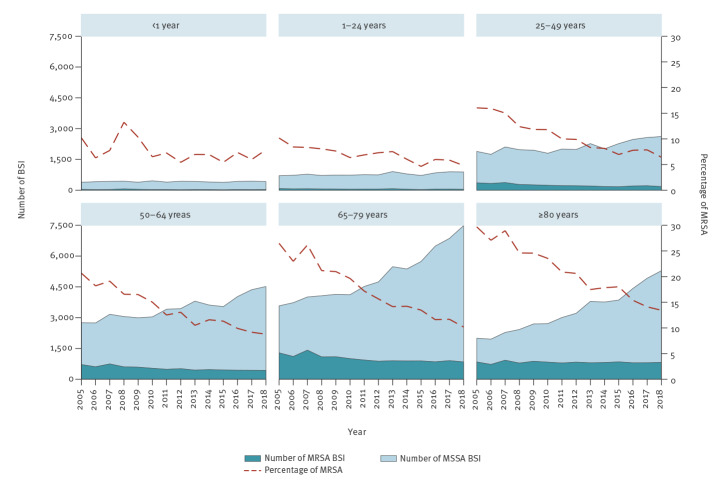
Bloodstream infections caused by meticillin-resistant *Staphylococcus aureus* and meticillin-susceptible *S. aureus*, by age group*,* EU/EEA, 2005–2018 (n = 249,550)

BSI: bloodstream infections; EEA: European Economic Area; EU: European Union; MRSA: meticillin-resistant *Staphylococcus aureus*; MSSA: meticillin-susceptible *S. aureus*.

Between 2005 and 2018, the MRSA percentage decreased significantly in all age groups (p = 0.003 for infants, age < 1 year; p < 0.001 for all other age groups) (Figure 2). During the same period, the number of MRSA BSI decreased significantly in all age groups except for patients 80 years and older, while the number of MSSA increased significantly in all age groups except for infants. Comparing 2005 with 2018, the number of MRSA BSI in the six age groups (< 1 year, 1–24 years, 25–49 years, 50–64 years, 65–79 years and ≥ 80 years) decreased by 18%, 44%, 50%, 39%, 34% and 3%, respectively. Conversely, the number of MSSA BSI increased by 9%, 26%, 38%, 64%, 109% and 164%, respectively.

Results from 285 laboratories in 25 EU/EEA countries, that consistently reported data during 2005 to 2018 (dataset B).

**Figure 3:**
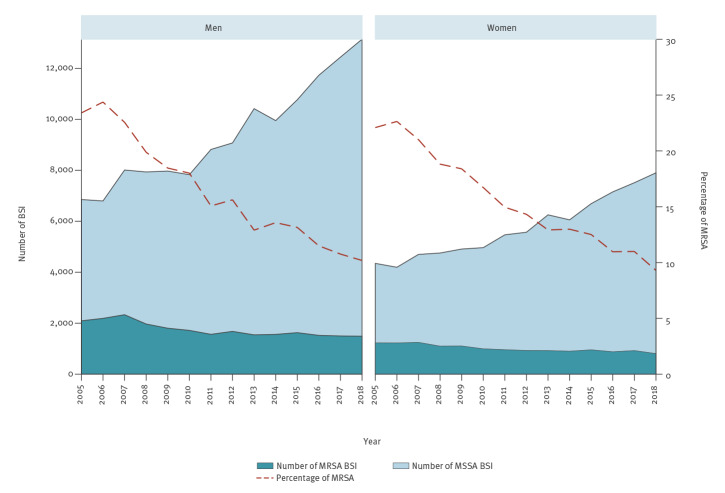
Bloodstream infections caused by meticillin-resistant and meticillin-susceptible *Staphylococcus aureus*, by sex, EU/EEA, 2005–2018 (n = 251,068)

BSI: bloodstream infections; EEA: European Economic Area; EU: European Union; MRSA: meticillin-resistant *Staphylococcus aureus*; MSSA: meticillin-susceptible *S. aureus*.

The sex-specific MRSA percentages decreased from 22.1% in 2005 to 9.3% in 2018 in women, and from 23.4% in 2005 to 10.2% in 2018 in men (Figure 3). During the same period, the number of MRSA BSI decreased while the number of MSSA increased both in women and in men; all trends were statistically significant (p < 0.001). Comparing 2005 with 2018, the number of MRSA BSI decreased by 34% and 29% in women and men, respectively. Conversely, the number of MSSA BSI increased by 82% and 92% in women and men, respectively.

Results from 285 laboratories in 25 EU/EEA countries, which consistently reported data during 2005 to 2018 (dataset B).

**Figure 4:**
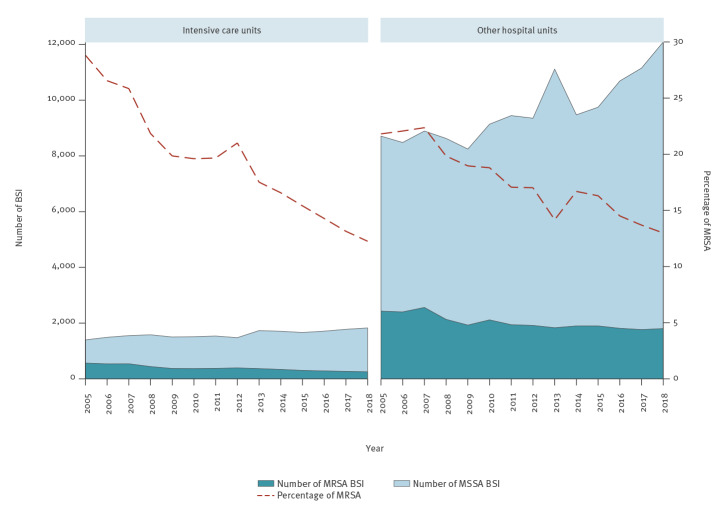
Bloodstream infections caused by meticillin-resistant and meticillin-susceptible *Staphylococcus aureus*, by type of hospital unit, EU/EEA, 2005–2018 (n = 191,509)

BSI: bloodstream infections; EEA: European Economic Area; EU: European Union; MRSA: meticillin-resistant *Staphylococcus aureus*; MSSA: meticillin-susceptible *S. aureus*.

The percentage of MRSA in ICU showed a linear decrease (p < 0.001) from 28.8% in 2005 to 12.3% in 2018 as the combined result of a decrease in the number of MRSA BSI (−55%) and an increase in the number of MSSA BSI (+31%) (Figure 4). The percentage of MRSA also decreased in BSI from other hospital units (from 21.8% in 2005 to 13.0% in 2018), but with a less pronounced decrease in the number of MRSA BSI (−26%) than for ICU and a similar increase in the number of MSSA BSI (+39%). The trends in the number of MRSA and MSSA BSI were statistically significant both in ICU and in other hospital units (p < 0.001).

Results from 285 laboratories in 25 EU/EEA countries, which consistently reported data during 2005 to 2018 (dataset B).

When restricting data to outpatients (19,684 BSI), we observed a significant linear decrease in the percentage of MRSA (from 18.5% in 2005 to 10.6% in 2018; p < 0.001). There was a significant increase in both the number of MSSA BSI (+199% from 2005 to 2018; p < 0.001) and the number of MRSA BSI cases (+57% from 2005 to 2018; p < 0.001) (Supplementary Figure S1).

The missing data for the variables ‘hospital unit’, age and sex were 25.9%, 3.4% and 2.9%, respectively (Supplementary Table S4). The BSI with missing information on the hospital unit showed MRSA percentages significantly lower than average, while those with missing data for age or sex showed MRSA percentages above average.

## Discussion

Data from EARS-Net showed a statistically significant increase in the number of *S. aureus* BSI in the EU/EEA between 2005 and 2018. This trend is supported by other published studies [17-20], which indicates that the health burden of BSI caused by *S. aureus* is growing in Europe.

Our analysis showed that the increase in *S. aureus* BSI was mainly the result of an increase in the number of MSSA BSI, while the number and the percentages of MRSA BSI decreased overall for the EU/EEA during the same period. As a result, the MSSA-to-MRSA ratio increased both when considering all *S. aureus* BSI reported to EARSS/EARS-Net during the study period (dataset A) or when restricting the analysis to only those laboratories which consistently reported data during the same period (dataset B). These trends were confirmed after adjusting for the increasing number of blood culture sets sampled during the study period.

Our results support the presence of the so-called Boyce effect, based on the assumption that MRSA and MSSA do not compete for the same ecological niche and that MRSA does not necessarily replace MSSA or vice versa [5-7]. Such a phenomenon can lead to apparently paradoxical trends characterised by a decrease in the percentage of MRSA and a general increase in the number of *S. aureus*, mostly MSSA, infections.

The ratio between MRSA and MSSA varied depending on patient characteristics. Stratification by age group showed that in younger patients, the decrease in the percentage of MRSA was smaller and mainly caused by the decrease in the number of MRSA BSI. Conversely, in the older age groups, the percentage of MRSA showed more pronounced decreasing trends, derived from the increase in the number of MSSA BSI. We also noted decreasing trends in MRSA percentages associated with a decrease in MRSA BSI combined with an increase in MSSA BSI when we stratified the analysis by sex and by hospital unit; these effects were more pronounced in BSI in men and in ICU patients. In outpatients on the other hand, the decrease in the percentage of MRSA was due to a larger increase in MSSA compared with MRSA.

The reasons for the increasing number of MSSA BSI in our data are likely to be multiple. An ageing population may affect the temporal trend of *S. aureus* infections, related to the increased risk of infection in the elderly [17]. As a consequence, a larger number of BSI could be expected, owing to the absolute growth of the elderly population in the EU/EEA during the study period. However, ageing cannot alone explain the observed increase in the number of MSSA BSI since the percentage shift of the general population from under 65 to over 65 years was estimated at only around 3% during the period from 2005 to 2018 [16]. Another explanation could be an increase in the use of blood culture sets as a result of diagnostic stewardship efforts during our study period, which may have improved the ascertainment of BSI cases [15]. Still, the increase in the number of MSSA BSI was statistically significant even after adjusting for the higher blood culture set rates. It is, however, possible that following a wider use of blood culture sets, the proportion of MRSA BSI ascertained by culture could have increased over time, leading to an underestimation of the real reduction in MRSA BSI during the study period.

Differences in MRSA and MSSA trends have been partly explained elsewhere by differences in their respective characteristics and modes of transmission, with MRSA being mainly a hospital pathogen showing lower genotypic diversity than MSSA [21-23]. A successful MSSA clone was described as having specific dynamics of colonisation, persistence and transmission, thus allowing its international spread although it is not multidrug-resistant [24]. Another noteworthy element is the possible increasing role of MSSA as a healthcare-associated pathogen, especially for community-onset but healthcare-associated BSI acquired in places such as wound care clinics, nursing homes and dialysis centres [22,25]. Information on whether a BSI was healthcare-associated is not available from EARS-Net, as it performs laboratory-based surveillance that does not collect clinical or epidemiological data on infection. Finally, interventions based on screening and isolation of positive patients have focused on MRSA, while to control MSSA, it is necessary to act effectively on non-specific measures such as hand hygiene [26].

Our study has several limitations. The ascertainment of BSI cases depends on the propensity to perform blood cultures [27]. Because of the response to empiric therapy with first-line antimicrobials, MSSA BSI are less likely to be ascertained when there is a low propensity to perform blood cultures which are reserved for cases such as treatment failure [6]. Therefore, an increasing blood culture rate could lead to a more pronounced increase in the ascertainment of BSI caused by MSSA than those caused by MRSA [6,17,27]. The increasing propensity to perform blood cultures in the period from 2005 to 2018, as observed in the EARS-Net dataset, makes it more difficult to interpret trends. However, given that the increase in the number of MSSA BSI over time was confirmed even after adjusting for the blood culture set rate, this confounding factor would only partially explain the observed trend for MSSA BSI, which we therefore considered real. This result is further supported by the data from outpatients, a context in which the implementation of stewardship activities promoting sampling of blood cultures is less likely and where the increase in MSSA BSI was greater than the average increase for the whole study sample.

Another potential issue that could have affected the results is the general tendency to centralise laboratories in large units, leading to a possible increase over time in the coverage of the laboratories considered in dataset B. However, in such a situation we would expect an increasing trend for both MRSA and MSSA BSI, since the variation in the number of reported MRSA and MSSA BSI should be proportional to the catchment population. Therefore, based on our results, it is unlikely that the coverage of laboratories that consistently reported data to EARS-Net increased substantially during the study period.

Finally, this analysis was based on routine laboratory data collected over a long time period. Records were sometimes incomplete, reducing the number of BSI that could be included in the analyses. For example, the percentage of missing data for the variable ‘hospital unit’ was 26%. Results by hospital unit were more representative of the countries with MRSA percentages above the average, since these countries had fewer missing data for the ‘hospital unit’ variable. The small percentage (3%) of missing data for sex and age is not likely to have biased our results.

### Conclusions

MRSA represents a known and major public health problem, and much attention has been given to the surveillance and prevention of MRSA infections. In the EU/EEA, the number of MRSA BSI has decreased significantly overall, but was still increasing in several EU/EEA countries during the period 2005 to 2018. The decrease in the percentage of MRSA among *S. aureus* BSI was mainly due to the increasing number of MSSA BSI overall and in most of the 25 EU/EEA countries that consistently reported data during the period 2005 to 2018. This increasing trend in the number of MSSA BSI in the EU/EEA needs further attention since MSSA BSI have high case fatality rates and there are still issues regarding their optimal treatment The seemingly conflicting results highlight the need to improve surveillance of AMR by combining data on AMR percentages with data on the number and incidence of infections. For EARS-Net, it would therefore be important to supplement the routine outputs, that currently only provide AMR percentages, with additional information on the number of infections caused by antimicrobial-resistant and -susceptible pathogens and possibly include an estimated incidence of infections. Further studies at local, national or supranational level, based on different data sources, are necessary to answer the questions raised by our results, in particular to identify all the factors associated with the described trends in MSSA and MRSA BSI. 

## Supplementary Data

248000 Supplement
