# Supplementary material for: Staphylococcus aureus bloodstream infections: diverging trends of meticillin-resistant and meticillin-susceptible isolates, EU/EEA, 2005 to 2018
Source: Euro Surveill. 2021 Nov 18;26(46):2002094. doi: 10.2807/1560-7917.ES.2021.26.46.2002094 (PMC8603406; doi:10.2807/1560-7917.ES.2021.26.46.2002094)
Supplement: Supplement [file 20-02094_HOGBERG_Supplement.pdf]

Supplementary material

This supplementary material is hosted by *Eurosurveillance* as supporting information alongside the article *A large increase in meticillin-susceptible Staphylococcus aureus bloodstream infections mostly explains the decrease in the percentage of meticillin-resistant S. aureus (MRSA) in the EU/EEA, 2005-2018*, on behalf of the authors, who remain responsible for the accuracy and appropriateness of the content. The same standards for ethics, copyright, attributions and permissions as for the article apply. Supplements are not edited by *Eurosurveillance* and the journal is not responsible for the maintenance of any links or email addresses provided therein.

Table S1. Number of *Staphylococcus aureus* bloodstream infections (BSIs), EU/EEA, 2005-2018: total, resistant to meticillin (MRSA) and susceptible to meticillin (MSSA), 2005-2018. Results from all laboratories (dataset A) and laboratories consistently reporting during the study period (dataset B)

Dataset	Countries (n)	Laboratories (n)	*S. aureus* BSIs (n)		
			Total	MRSA	MSSA
A	30	1,295	573,951	96,918	477,033
B	25	285	258,448	41,033	217,415

Table S2. Trends of *Staphylococcus aureus* bloodstream infections (BSIs), by country: number of BSIs caused by meticillin-susceptible *S. aureus* (MSSA); number of BSIs caused by meticillin-resistant *S. aureus* (MRSA) and percentage of MRSA. Results from 285 laboratories in 25 EU/EEA countries, which consistently reported data during 2005-2018 (dataset B)

**Country**	**Significance and direction of trends**		
	MSSA (n)	MRSA (n)	MRSA (%)
Austria	↑	–	↓
Belgium	↑	↓	↓
Bulgaria	↑	↓	↓
Cyprus	↑	↑	–
Czech Republic	↑	↑	–
Denmark	↑	↑	↑
Estonia	↑	–	–
Finland	↑	↑	–
France	↑	↓	↓
Greece	↑	↓	↓
Hungary	↑	↑	–
Iceland	–	–	–
Ireland	↑	↓	↓
Italy	↑	↑	↓
Latvia	↑	–	↓
Luxembourg	↑	–	↓
Malta	↑	↓	↓
Netherlands	↑	–	–
Norway	↑	↑	↑
Portugal	↑	↑	↓
Romania	↑	↑	–
Slovenia	↑	↑	↑
Spain	–	–	–
Sweden	↑	↑	↑
United Kingdom	↑	↓	↓

↓ *(statistically significant decreasing trend);* ↑ *(statistically significant increasing trend); – (no statistically significant trend)*

Table S3. Weighted average blood culture set rates for countries included in dataset B, by year, 2005-2018

**Year**	**Average rate based on all available data***		**Average rate based on selected data****	
	N. countries	*Blood culture sets/1000 patient-days*	*N. countries*	*Blood culture sets/1000 patient-days*
2005	23	35.8	13	33.1
2006	23	36.9	13	36.6
2007	24	36.3	13	36.2
2008	24	41.0	13	41.7
2009	23	36.7	13	36.5
2010	21	40.3	13	37.9
2011	22	46.3	13	42.3
2012	18	47.2	13	43.3
2013	20	53.9	13	49.7
2014	20	65.8	13	64.1
2015	21	69.2	13	67.0
2016	19	71.3	13	66.7
2017	23	77.7	13	68.7
2018	21	81.7	13	71.8

** Countries with blood culture set rates available for at least one year during 2005-2018*

*** Countries with blood culture set rates available for all years*

Table S4. Number of missing values for patient characteristics variables. Data from 285 laboratories in 25 EU/EEA countries, which consistently reported data during 2005-2018 (dataset B)

**Variable**	**Missing values**	
	(n)	(%)
Sex	7,380	2.9
Age	8,898	3.4
Hospital unit	66,939	25.9

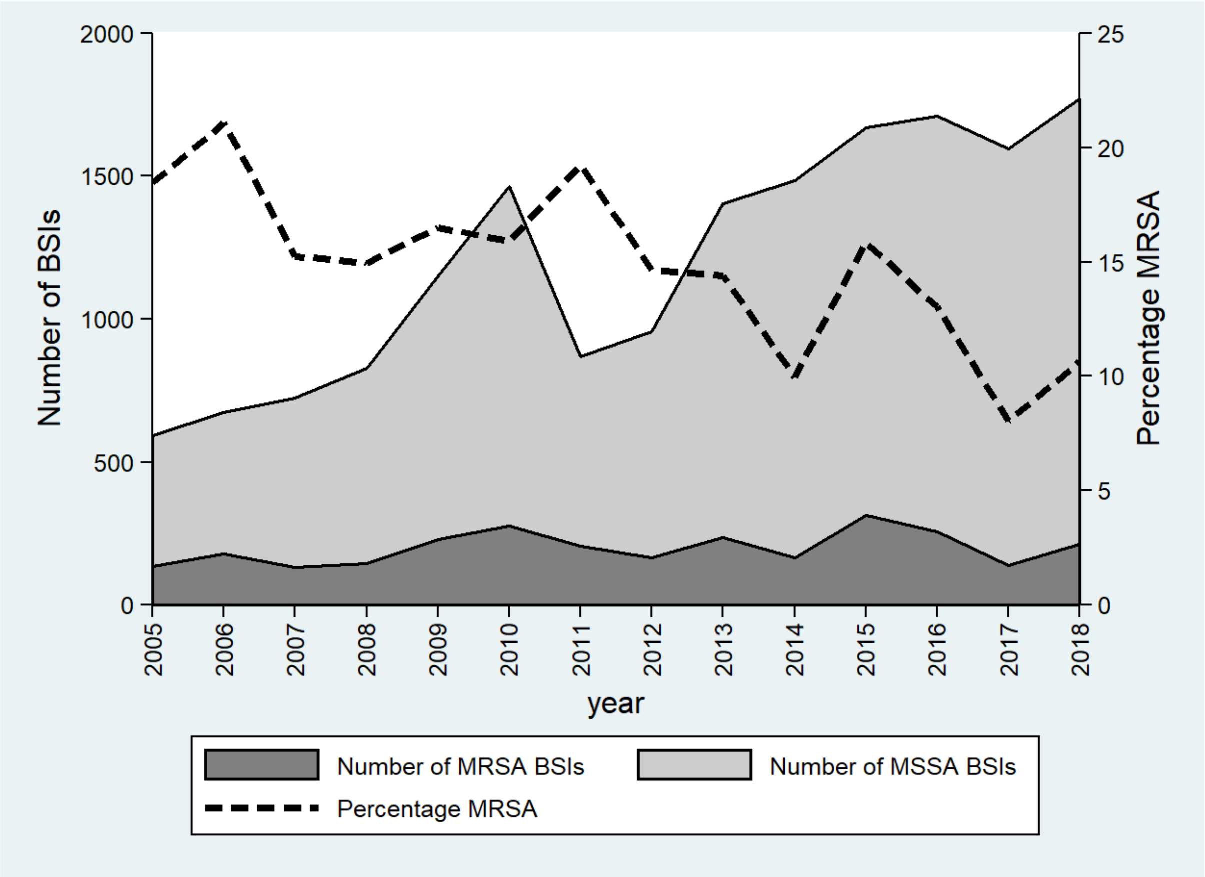

Figure S1*.* Number and percentage of bloodstream infections (BSIs) caused by meticillin-resistant *Staphylococcus aureus* (MRSA), number of BSIs caused by meticillin-susceptible *S. aureus* (MSSA) in outpatients. Results from 285 laboratories in 25 EU/EEA countries, which consistently reported data during 2005-2018 (dataset B).
